# Decreased Compressional Sound Velocity Is an Indicator for Compromised Bone Stiffness in X-Linked Hypophosphatemic Rickets (XLH)

**DOI:** 10.3389/fendo.2020.00355

**Published:** 2020-06-09

**Authors:** Adalbert Raimann, Sarah N. Mehany, Patricia Feil, Michael Weber, Peter Pietschmann, Andrea Boni-Mikats, Radka Klepochova, Martin Krššák, Gabriele Häusler, Johannes Schneider, Janina M. Patsch, Kay Raum

**Affiliations:** ^1^Division of Pediatric Pulmonology, Allergology and Endocrinology, Department of Pediatrics and Adolescent Medicine, Comprehensive Center for Pediatrics, Medical University of Vienna, Vienna, Austria; ^2^Division of General and Pediatric Radiology, Department of Biomedical Imaging and Image-guided Therapy, Medical University of Vienna, Vienna, Austria; ^3^Division of Pediatric Surgery, Department of Surgery, Medical University of Vienna, Vienna, Austria; ^4^Department of Pathophysiology and Allergy Research, Medical University of Vienna, Vienna, Austria; ^5^Department of Biomedical Imaging and Image-guided Therapy, The High Field MR Centre, Vienna, Austria; ^6^Division of Endocrinology and Metabolism, Department of Medicine III, Medical University of Vienna, Vienna, Austria; ^7^Christian Doppler Laboratory for Clinical Molecular MR Imaging—MOLIMA, Vienna, Austria; ^8^Charité—Universitätsmedizin Berlin, Corporate Member of Freie Universität Berlin, Humboldt-Universität zu Berlin, and Berlin Institute of Health, BCRT - Berlin Institute of Health Center for Regenerative Therapies, Berlin, Germany

**Keywords:** XLH, axial transmission, hypophosphatemia, HR-pQCT, rickets, ultrasound, bone, rare disease

## Abstract

**Objectives:** To assess the diagnostic potential of bidirectional axial transmission (BDAT) ultrasound, and high-resolution peripheral quantitative computed tomography (HR-pQCT) in X-linked hypophosphatemia (XLH, OMIM #307800), a rare genetic disorder of phosphate metabolism caused by mutations in the *PHEX* gene.

**Methods:** BDAT bone ultrasound was performed at the non-dominant distal radius (33% relative to distal head) and the central left tibia (50%) in eight XLH patients aged between 4.2 and 20.8 years and compared to twenty-nine healthy controls aged between 5.8 and 22.4 years. In eighteen controls, only radius measurements were performed. Four patients and four controls opted to participate in HR-pQCT scanning of the ultradistal radius and tibia.

**Results:** Bone ultrasound was feasible in patients and controls as young as 4 years of age. The velocity of the first arriving signal (ν_FAS_) in BDAT ultrasound was significantly lower in XLH patients compared to healthy controls: In the radius, mean ν_FAS_ of XLH patients and controls was 3599 ± 106 and 3866 ± 142 m/s, respectively (−6.9%; *p* < 0.001). In the tibia, it was 3578 ± 129 and 3762 ± 124 m/s, respectively (−4.9%; *p* = 0.006). HR-pQCT showed a higher trabecular thickness in the tibia of XLH patients (+16.7%; *p* = 0.021).

**Conclusions:** Quantitative bone ultrasound revealed significant differences in cortical bone quality of young XLH patients as compared to controls. Regular monitoring of XLH patients by a radiation-free technology such as BDAT might provide valuable information on bone quality and contribute to the optimization of treatment. Further studies are needed to establish this affordable and time efficient method in the XLH patients.

## Introduction

X-linked hypophosphatemia (XLH, OMIM #307800) is a rare disorder of phosphate homeostasis with an estimated prevalence of 8–17 per million (1). Caused by a loss-of-function mutation in the *PHEX* (phosphate regulating endopeptidase homolog, X-linked) gene, upregulation of FGF23 expression leads to an inhibition of renal phosphate reabsorption and results in low serum phosphate levels and impaired 1-alpha-hydroxylase activity (2, 3). Despite x-chromosomal inheritance and heterozygosity in females, penetrance is reported to be 100% by 1 year of age in both sexes (4).

Key symptoms of the profound chronic hypophosphatemia are progressive bone deformities, which mostly occur at the age of weight bearing. Further, impaired longitudinal growth and disproportionate short stature impair quality of life in adult age (5, 6). In childhood, radiographic signs of rickets manifest as widening of the growth plates and metaphyseal flaring (7). Extra-skeletal ossifications in ligaments or at ligament attachment sites, called enthesopathies, may occur in later adulthood. Endodontic problems such as root infections and early loss of teeth are common among the XLH population (6). Different forms of hypophosphatemic rickets are often associated with muscle weakness, which is usually mild in XLH patients (8, 9). However, radiologic presentation and clinical phenotype are extremely variable and do not seem to be linked with genotype (10, 11).

Dysregulation of matrix regulation and impaired mechanical resistance due to chronic hypophosphatemia are causative for the long-term development of mobility impairing deformities of the lower extremity. Thus, skeletal imaging in pediatric XLH patients for the evaluation of the affection of the mineralizing matrix is highly valuable for initial work-up, monitoring of treatment as well as evaluation of surgical options. Clinical imaging is mostly based on radiographs and rickets severity scoring (RSS) as described and validated by Thacher et al. (12, 13). Due to the lack of quantitative tools, standardized but subjective RSS rating is considered as gold-standard for rachitic affection of bone. While this observer-dependent scoring of the affection of growth plates and adjacent mineralizing tissue has been validated in XLH Patients (13), surgical interventions are mostly performed in diaphyseal bone which is not rated by RSS. Surgical planning for the correction of limb deformities, axial deviations, or length calculations is commonly assessed by cross-sectional imaging such as computed tomography (CT) or magnetic resonance imaging (MRI). With these imaging modalities the mineralization phenotype can only be assessed indirectly by means of growth-plate abnormalities or deformities. Therefore, complementary information about tissue properties would be valuable in pre-surgery assessment in rachitic disorders such as XLH.

In XLH patients, dual-energy x-ray absorptiometry (DXA) studies have shown a tendency of higher mineralization in the axial skeleton and lower mineralization in the appendicular skeleton (6, 7, 14, 15). As a two-dimensional measurement of a three-dimensional structure, DXA only reflects areal bone mineral density (aBMD) (7). Moreover, DXA does not provide information on bone microarchitecture and compartment-specific BMD. In aBMD, a size artifact arises, where small bones seem to have lower BMD and large bones higher BMD. Considering the growth disturbances in XLH, DXA results have to be interpreted with caution (16). To account for this size artifact, Carter et al. proposed a calculation of bone mineral apparent density (BMAD) to estimate volumetric BMD (vBMD) (7, 17). BMAD can be calculated by mathematical equations using DXA aBMD results (7). Beck-Nielsen et al. reported that children and adults with XLH have elevated BMAD of the lumbar spine (7). However, Colares Neto et al. examined 37 children and adults with XLH stratified by age group and reported that mean aBMD was only elevated in adults (16). DXA interpretation in XLH patients remains complex and results may not accurately portray bone mineralization (7, 16). Factors such as anthropometric data or presence of enthesopathies can influence aBMD (6, 7).

High-resolution peripheral quantitative computed tomography (HR-pQCT) can be used to assess bone geometry, bone microarchitecture, and compartment-specific volumetric bone mineral density (vBMD) at the distal radius and the distal tibia (18). Until now, the literature on HR-pQCT in patients with XLH remains limited (16, 19). Colares Neto et al. examined bone mineral density and microarchitecture by HR-pQCT and found that XLH patients had lower trabecular vBMD and thus lower total vBMD, especially at the load-bearing distal tibia. The trabecular microarchitecture was significantly different in both the radius and the tibia with XLH patients having lower trabecular number, more trabecular separation, and higher trabecular network inhomogeneity (16). Shanbhogue et al. reported similar changes in trabecular microarchitecture (19).

Quantitative ultrasound (QUS) is increasingly being used to assess bone status beyond BMD (20, 21). Apart from its cost-effectiveness and convenience, a radiation-free method of bone assessment is desirable, especially in children. Most of the current QUS technologies focus on the measurement of cortical bone at radius, tibia, or phalanges, either in axial, or in through-transmission modes. Various applications of QUS in children are summarized in (22). These and other studies conducted in adults confirm that parameters derived from QUS measurements provide information that is, at least in part, independent of BMD. However, for many QUS modalities the associations between the derived parameters and bone properties are only poorly understood. During the last decade, much effort has been dedicated to the so-called bi-directional axial transmission (BDAT) modality. The bi-directional measurement corrects for probe inclination due to variable soft-tissue thickness. The velocity of the first-arriving signal (hereinafter simply called ν_FAS_) is directly linked to cortical tissue stiffness, cortical porosity (Ct.Po), and cortical thickness (Ct.Th) (23). It should be noted that the thickness dependence is related to the ratio of the acoustic wavelength to thickness, i.e., for low frequencies and thin cortical bone, the latter has a dominant effect on variations of ν_FAS_, whereas for higher frequencies and thicker cortical bone, ν_FAS_ becomes independent of Ct.Th variations. The most recent system uses this relationship by means of a spatiotemporal spectral analysis and some assumptions, such as a constant tissue stiffness, to obtain Ct.Th and Ct.Po at the radius and tibia of adult humans (24, 25).

This pilot study is the first to examine BDAT ultrasound in patients with XLH. The ν_FAS_ parameter was used as a marker for variations in cortical bone properties. As it is known that XLH leads to mineralization defects with many hypo-mineralized holes in the cortical tissue matrix, increased pore size, and higher porosity (26), we hypothesized that the combined effect of compromised tissue mineralization and increased porosity in XLH patients results in decreased compressional wave velocities, as measured by means of ν_FAS_ at 1 MHz. Secondary aims were to assess microarchitectural changes of trabecular and cortical bone by HR-pQCT in patients with XLH.

## Materials and Methods

### Subjects

This study was approved by the ethics committee of the Medical University of Vienna (Vote 2201/2015). We obtained written informed consent from all participants (or their guardians, depending on participant age), and assent from children above the age of 14 years.

Children above the age of four, adolescents, and young adults were included in this study. Diagnosis of XLH was confirmed in all patients either by genetic analysis or by biochemical testing in genetically verified familial cases. All patients were diagnosed and treated within the first 2 years of life (Table 1). Patient compliance was considered as sufficient in 7/8 patients by the treating physician. All XLH patients had blood draws as part of routine care. Serum calcium, serum phosphate, parathyroid hormone (PTH), vitamin D [25-(OH)D], calcitriol [1,25-(OH)2D], and alkaline phosphatase activity (ALP) were analyzed. In addition, urinary parameters such as calcium/creatinine ratio and the ratio of tubular maximum reabsorption of phosphate to glomerular filtration rate (TmP/GFR) were measured in XLH patients.

**Table 1:** Descriptive statistics of XLH patients.

		**XLH patients (*n* = 8)**	**Reference ranges**
Basic clinical parameters	Age (yr)	12.9 ± 5.8 [13.4]	
	Sex (f/m)	7/1	
	Body height (cm)	135.0 ± 23.3 [145.4]	
	Body height SDS	−2.2 ± 1.1 [−2.0]	
	BMI SDS	0.58 ± 1.06 [0.99]	
	Body ratio (sitting height/leg length) SD	1.7 ± 1.3 [1.65]	
Laboratory parameters	Serum Ca (mmol/L)	2.3 ± 0.1 [2.3]	2–12 y: 2.2–2.7; 12–18 y: 2.1–2.55
	Serum P (mmol/L)	0.88 ± 0.11 [0.9]	1–12 y: 1.0–2.3; > 12 y: 0.81–1.45
	PTH (pg/mL)	51.48 ± 20.08 [48.2]	15–65 pg/ml
	25D (nmol/L)	50.74 ± 22.60 [51.1]	> 50 nmol/L
	1,25D (pg/mL)	44.88 ± 18.19 [45]	19.9–79.3 pg/ml
	ALP (U/L)	309.13 ± 174.40 [360]	Age-specific according to (39)
Urinary parameters	Ca/Crea ratio	0.20 ± 0.28 [0.115]	<0.70 mmol/mmol
	TmP/GFR (mmol/L)	0.73 ± 0.08 [0.69]	1.15–2.44
Treatment	Phosphat (mg/kg KG/d)	40.25 ± 15.16 [40.06]	
	Calcitriol (ng/kg KG/d)	19.47 ± 12.62 [21.13]	
	Treatment duration (y)	11.63 ± 5.3	

*Values are expressed as mean ± SD [median]*.

As control group, healthy children, adolescents, and young adults (*n* = 4) were recruited from the Division of Pediatric Surgery, Department of Surgery, Medical University of Vienna. Additional QUS control data were available from volunteers measured at Charité-Universitätsmedizin Berlin (*n* = 26). In total, QUS data from 37 subjects (i.e., 8 XLH patients, 3 controls, data from 26 healthy volunteers) were included in this study. HR-pQCT scanning of the ultradistal radius and tibia was performed in four patients and four controls.

Exclusion criteria for the control group were chronic diseases that affect the motion apparatus, increased likelihood of fractures, bone deformities, chronic inflammatory diseases, malabsorption, metabolic diseases, or diseases of the muscle. Additional exclusion criteria for both the XLH and the control group included bilateral lower leg or forearm fractures, and pregnancy.

### Quantitative Ultrasound (QUS)

**Figure 1:**
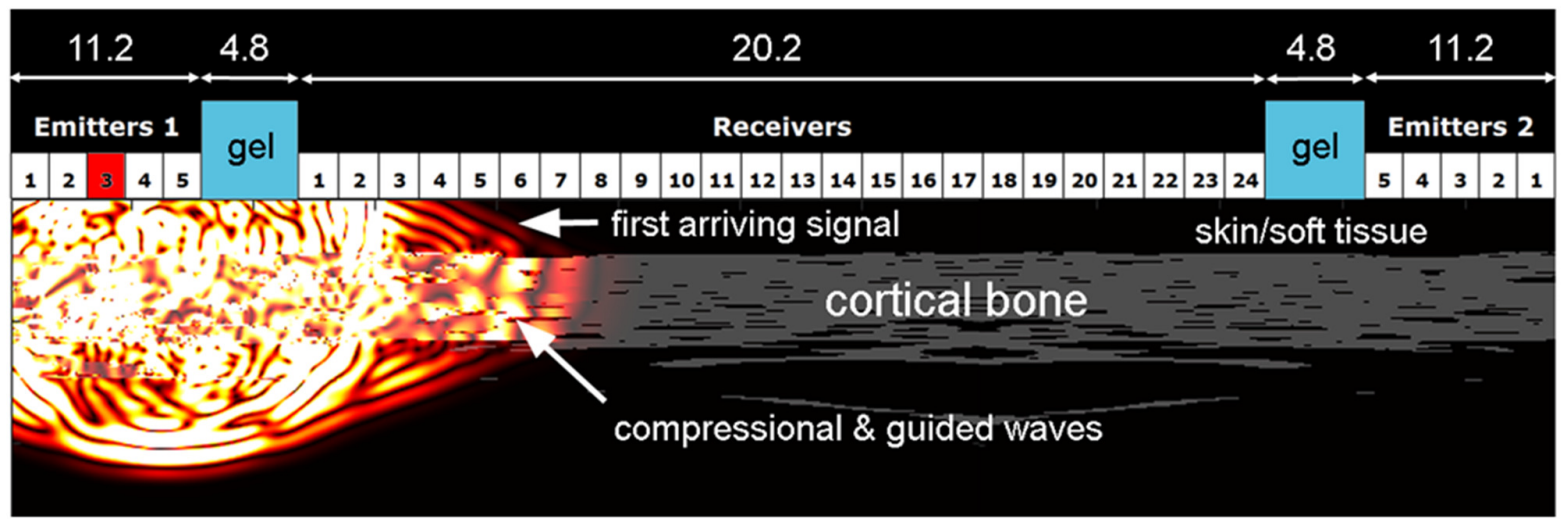
Principle of bi-directional axial ultrasound transmission. The ultrasound transducer consists of two emitter arrays and one receiver array separated by gel filled gap regions (dimensions are given in mm). The numerical sound propagation simulation shows an ultrasound pulse emitted at element 3 of emitter array 1, which propagates through skin and soft tissue into the bone. One part of the wave is transmitted into the medullary canal and other parts propagate as compressional and dispersive guided waves in the axial bone direction through the cortical shell. These waves leak acoustic waves back into the soft tissue which are detected by the central receiver array.

**Figure 2:**
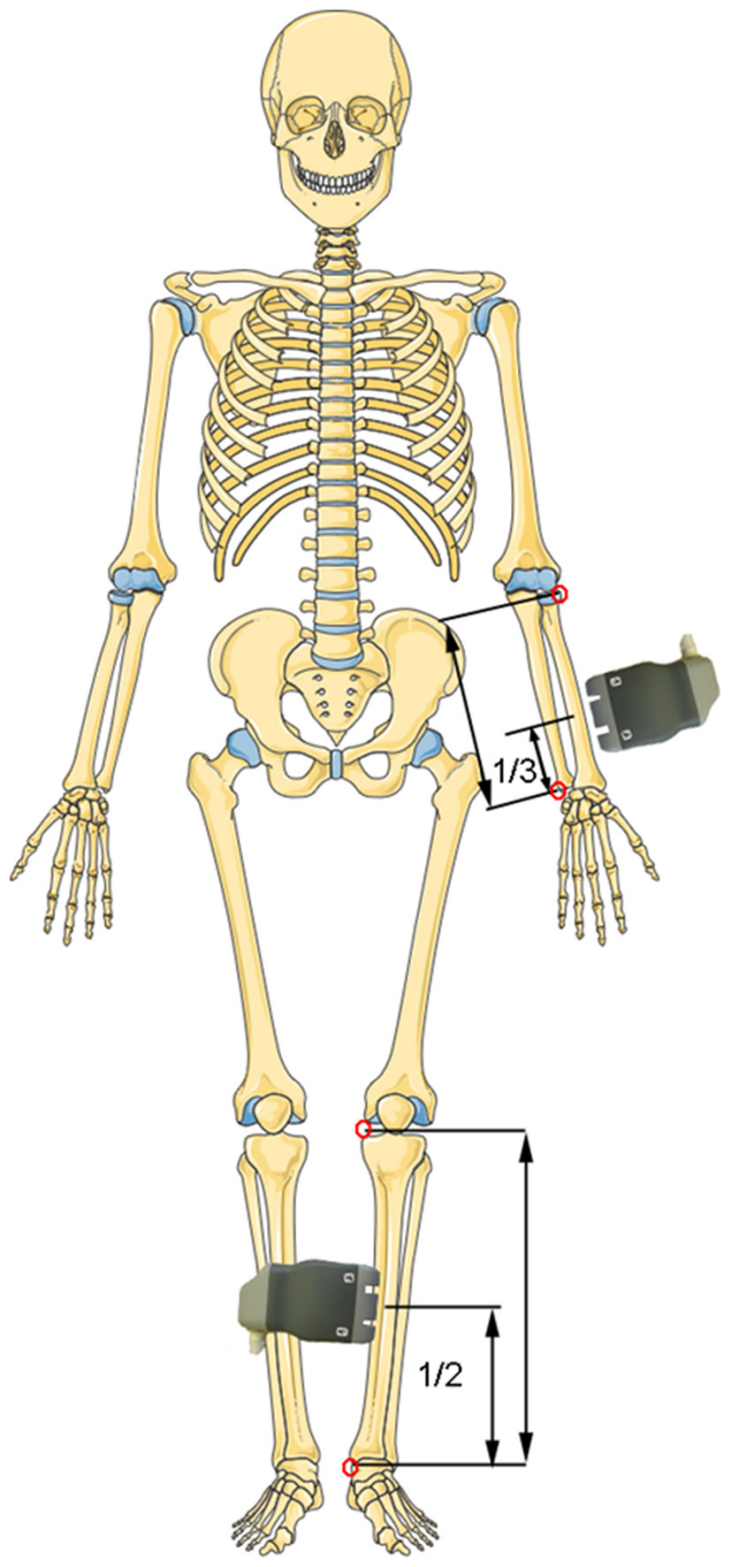
Ultrasound measurement locations. The landmarks used for the identification of the longitudinal positions are marked by red dots. (Adapted from Servier Medical Art by Servier under a Creative Commons Attribution 3.0 Unported License).

Ultrasound measurements were performed using a prototype system (BDAT Ultrasonic Module, serial number 001, software version 3.1, CETU Althaïs Technologies, Tours, France), as described in (27). Briefly, the system consists of a custom array transducer (Figure 1) with 2 × 5 transmitter elements on each side and 24 central receiver elements (Vermon, Tours, France). The transmit elements are subsequently excited with short positive signals, which resulted in the emission of broadband pulses of about 0.5–1.5 MHz. For each excitation, the transmitted signals were recorded simultaneously with all receiver elements. Each measurement cycle was repeated 100 times. During the measurement the probe was slightly moved, to ensure the acquisition under variable probe orientations relative to the bone. The measurements were performed at the non-dominant distal radius (33% relative to distal head) and the central left tibia (50%). At the tibia the probe was centered at the medial side (M) and at the radius, the probe was centered at the postero-lateral side (Figure 2). In case of a local fracture or fracture history of the non-dominant radius or left tibia, the contralateral side was scanned. If a measurement of ν_FAS_ has been repeated, the median of all measurements was calculated. For the evaluation of QUS, additional control patients from Berlin have been included in the data analysis. Note that in eighteen out of these twenty-six controls, only measurements at the radius were made. As a result, 8 children and adolescents with XLH (4–20 years old) and 29 healthy age-matched controls (5–22 years old) have been included. Independent of the recruitment site, all subjects were measured with the same ultrasound device by two experienced operators (KR and JS).

#### QUS Data Analysis

**Figure 3:**
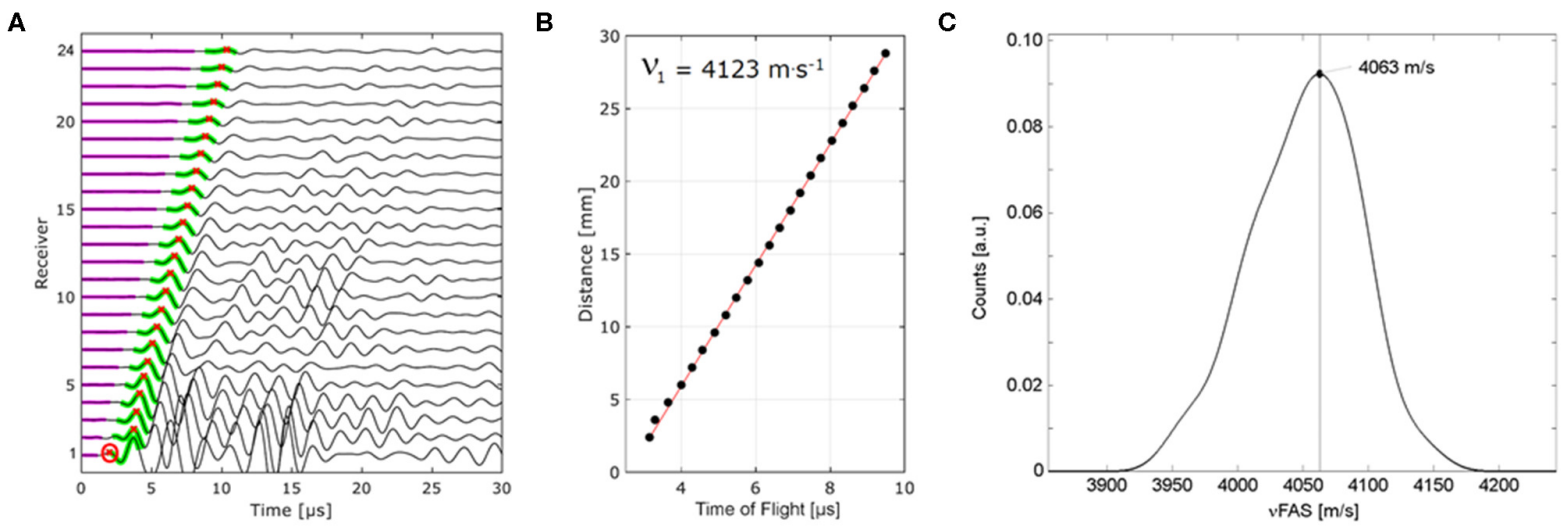
Ultrasound signals recorded at the receiver array after the excitation of a signal from one emitter **(A)**. For each receiver element, the first arriving signal exceeding the noise floor (magenta) is time-gated (green) and the time-of-flight of the first peak (red crosses) is detected. Pulse travel times linearly increasing with emitter-receiver distance are used to calculate the propagation velocity in this direction **(B)**. Other signals [the encircled signal of receiver channel 1 in **(A)**], were automatically excluded. From the distribution of all measured velocities, the peak position was used for further evaluation.

The estimation of the velocity of the first arriving signal has been described in detail (28). The first signal arriving at a receiver element can be a signal (i) propagating directly from emitter to receiver through soft tissue with a velocity of approximately 1540 m/s, (ii) coupled into cortical bone as a non-dispersive compressional wave with a velocity > 3,500 m/s, or (iii) coupled into the cortical shell as a dispersive guided wave (Figure 1). We have developed an optimized automated algorithm that recognizes receiver elements detecting wave propagation only through soft tissue and excluded those from subsequent ν_FAS_ calculation (Figure 3A). The velocity in one propagation direction was determined from the linear slope between arrival time and emitter-receiver distance (Figure 3B). The harmonic mean of the velocities in both propagation directions was calculated to compensate for variable soft-tissue thickness along the propagation path. From the distribution of all measured velocities, the one occurring with the highest frequency (peak position) was used for further analysis (Figure 3C). The inter-operator *in-vivo* precision for this measurement is in the order of 0.5% (28).

### HR-pQCT Imaging

To assess volumetric bone mineral density, bone geometry, and bone microarchitecture of the ultra-distal extremities, we used a clinical HR-pQCT system (XtremeCT I, Scanco Medical, Brütisellen, Switzerland). The non-dominant ultradistal radius and left ultradistal tibia were scanned. After an anteroposterior scout projection, a reference line was placed at the physis or the physis remnant. In XLH, the reference line was placed at the nadir of the cupped physis. Cupping in skeletally immature children with XLH was only mild in our patient cohort (Thacher Grade 1) (12). In between the reference line and the scan volume, we used a fixed distance of 2 mm. Each scan volume was 9 mm long. The standard *in-vivo* protocol as described in the literature used the following settings: 60kVP, 900 μA, 100 ms integration time (18, 29). The matrix size was 1536 × 1536, and the nominal resolution 82 μm.

In case of a local fracture or fracture history of the non-dominant radius or left tibia, the contralateral side was imaged. Scan time was ~3 min per site and the effective dose in radius and tibia 3 μSv, respectively. If unacceptable motion occurred, scans were repeated once.

#### Trabecular Bone Analysis

The standard evaluation software of Scanco Medical XtremeCT has been used to evaluate trabecular parameters including trabecular bone volume fraction (BV/TV), trabecular number (Tb.N), trabecular thickness (Tb.Th), trabecular spacing (Tb.Sp), and trabecular network inhomogeneity (Tb.1/N.SD). The precisions for these parameter estimations have been reported in the range between 1.0 and 4.4% (18).

#### Cortical Bone Analysis

For extended cortical analysis a method developed by Burghardt et al. has been employed (30–32). Parameters evaluated were cortical BMD (Ct.BMD), cortical thickness (Ct.Th), cortical pore volume (Ct.PoV), cortical porosity (Ct.Po), mean pore diameter (Po.Dm), and standard deviation of mean pore diameter (Dm.SD). The precisions for the structural parameter estimations have been reported to be in the range between 0.6 and 0.92% (30).

### Statistical Analysis

IBM SPSS Version 24 was used for statistical analysis. Data distribution was explored by inspection of histograms and boxplots. A Lilliefors test was used to test if data were normally distributed. Linear regression analysis and Pearson's correlation coefficients were used to assess the age dependency of ν_FAS_. For normally distributed values, mean and standard deviation were calculated. For values with non-normal distribution, the median and percentiles were calculated. An unpaired student's t-test was used to test for differences between XLH patients and controls. Statistical significance was defined as *p* ≤ 0.05.

## Results

### Subject Characteristics

In total, QUS data from 37 subjects were included in this study. There was no significant difference in the mean age of XLH patients and QUS controls (12.9 ± 5.8 vs. 15.9 ± 4.1 years; *p* = 0.10). Sex ratio was 1:8 males in XLH patients compared to 5:29 male QUS controls. All XLH patients received conventional treatment with phosphate and activated vitamin D since early childhood. For detailed patient XLH characteristics see Table 1. The HR-pQCT controls were 9.9–22.8 years old (mean age 15.6 ± 4.7 years) and consisted of three females and one male. The XLH patients that participated in HR-pQCT were 14.4 ± 3.5 years old compared to 15.6 ± 4.7 years for controls. XLH patients differed from the reference population regarding height (mean z-score −2.2) according to Austrian reference values (33). Sitting height to leg length ratio was higher than in the reference population (mean z-score 1.7).

### Quantitative Ultrasound (QUS)

**Figure 4:**
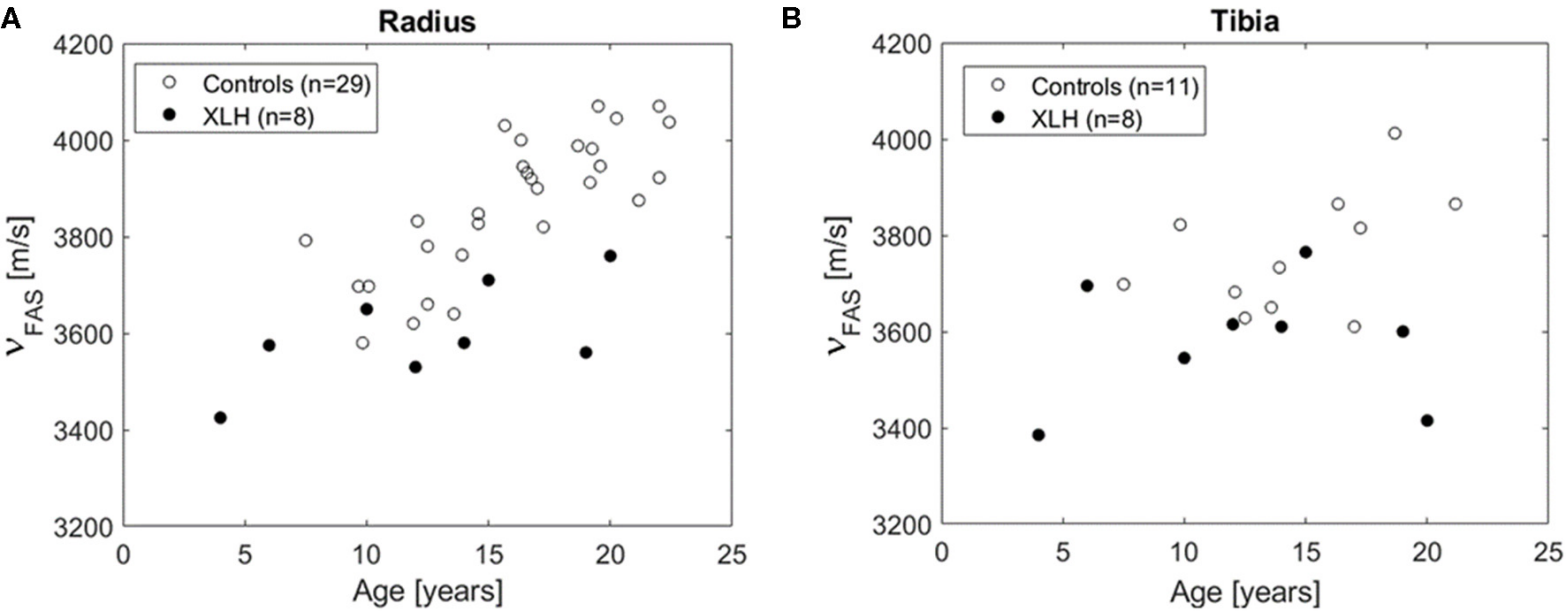
Results of quantitative ultrasound at the radius **(A)** and the tibia **(B)**.

In total, eight XLH patients and 29 controls participated in the QUS assessment. All XLH patients and 11 controls were measured at radius and tibia. From 18 controls, no tibia data were available. The ν_FAS_ values were normally distributed. There was a positive association of ν_FAS_ with age in the radius (*R* = 0.74, *p* < 0.001), but not in the tibia (Figure 4). Significant differences in ν_FAS_ between XLH patients and controls were observed in both, radius and tibia: In the radius, mean ν_FAS_ of XLH patients and controls was 3599 ± 106 and 3866 ± 142 m/s, respectively (−6.9%; *p* < 0.001). In the tibia, it was 3578 ± 129 and 3762 ± 124 m/s, respectively (−4.9%; *p* = 0.006). As can be seen in Figure 4, ν_FAS_ remained consistently lower in XLH patients across the age range of our study population.

### HR-pQCT

**Figure 5:**
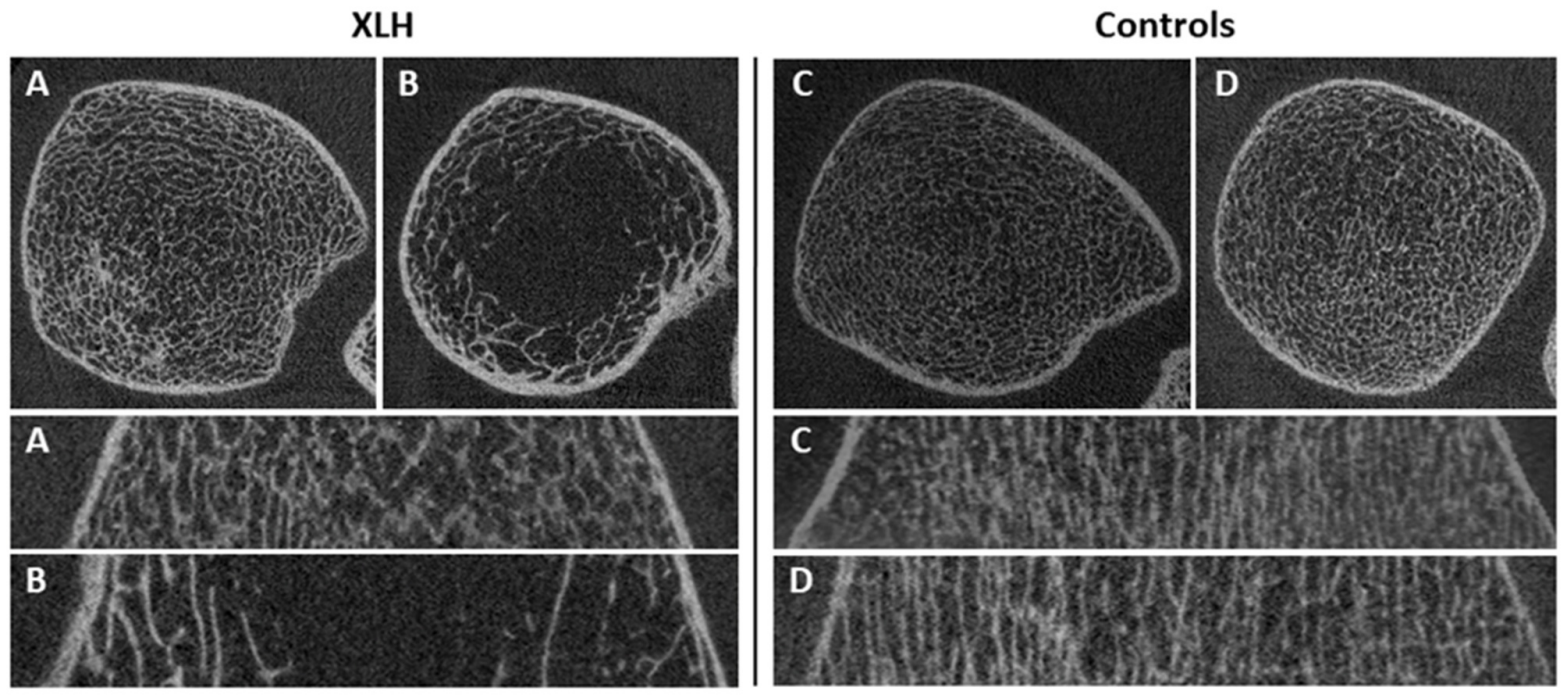
Representative axial and coronal HR-pQCT images of the ultradistal tibia of XLH patients (*n* = 2; **A,B**) compared to healthy controls (*n* = 2; **C,D**). Images marked with the same letter belong to the same individual.

Four XLH patients and four controls opted to participate in HR-pQCT scanning. Figure 5 shows HR-pQCT images of two XLH patients and two controls in the axial and reconstructed coronal plane. Mean values and standard deviations can be found in Table 2. Trabecular thickness (Tb.Th) in the tibia was significantly higher in XLH patients than in controls (+16.7%; *p* = 0.021). In the radius, Tb.Th was also increased in XLH patients, however not statistically significant (+33.3%; *p* = 0.118). In both the radius and tibia, XLH patients exhibited lower mean trabecular number (Tb.N), higher mean trabecular spacing (Tb.Sp) and higher mean trabecular network inhomogeneity (Tb.1/N.SD), though not statistically significant. Cortical thickness (Ct.Th) in the tibia showed a tendency to be higher in XLH patients than in controls (+18.8%; *p* = 0.061). Paired data of patients undergoing both HR-pQCT and QUS can be found in Figure S1. Because of the low number of paired data (*n* = 7) no statistical analysis was performed.

**Table 2:** Results of HR-pQCT in XLH patients and controls.

	**Controls (*n* = 4)**	**XLH patients (*n* = 4)**	***p***	**Relative difference compared to controls (%)**
**ULTRADISTAL RADIUS**				
**Basic HR-pQCT measures**				
Ct.BMD (mg HA/cm3)	676 ± 105	651 ± 98	0.742	−3.7
Ct.Th (mm)	0.72 ± 0.10	0.74 ± 0.10	0.809	2.8
BV/TV	0.13 ± 0.03	0.15 ± 0.03	0.384	15.4
Tb.N (1/mm)	2.12 ± 0.45	1.98 ± 0.30	0.611	−6.6
Tb.Th (mm)	0.06 ± 0.01	0.08 ± 0.02	0.118	33.3
Tb.Sp (mm)	0.43 ± 0.12	0.44 ± 0.09	0.888	2.3
Tb.1/N.SD (mm)	0.17 ± 0.06	0.19 ± 0.06	0.607	11.8
**Porosity**				
Ct.PoV (mm3)	1.36 ± 0.83	0.54 ± 0.34	0.115	−60.3
Ct.Po (%)	0.28 ± 0.17	0.17 ± 0.18	0.403	−33.3
Po.Dm (mm)	0.15 ± 0.03	0.14 ± 0.03	0.591	−6.7
Dm.SD (mm)	0.07 ± 0.03	0.06 ± 0.03	0.665	−14.3
**ULTRADISTAL TIBIA**				
**Basic HR-pQCT measures**				
Ct.BMD (mg HA/cm3)	651 ± 80	692 ± 79	0.499	6.2
Ct.Th (mm)	0.69 ± 0.10	0.82 ± 0.05	0.061	18.8
BV/TV	0.15 ± 0.02	0.13 ± 0.04	0.424	−13.3
Tb.N (1/mm)	2.33 ± 0.41	1.72 ± 0.60	0.147	−26.2
Tb.Th (mm)	0.06 ± 0.00	0.07 ± 0.00	0.021*	16.7
Tb.Sp (mm)	0.38 ± 0.08	0.59 ± 0.34	0.252	55.3
Tb.1/N.SD (mm)	0.15 ± 0.04	0.31 ± 0.25	0.245	106.7
**Porosity**				
Ct.PoV (mm3)	4.10 ± 3.01	3.32 ± 4.87	0.793	−19.0
Ct.Po (%)	0.55 ± 0.32	0.38 ± 0.54	0.592	−33.3
Po.Dm (mm)	0.15 ± 0.01	0.18 ± 0.03	0.092	20.0
Dm.SD (mm)	0.06 ± 0.01	0.10 ± 0.05	0.174	66.7

Values are expressed as mean ± SD. Bold font* indicates a significant difference.

## Discussion

The main finding of this study was that the velocity of the first arriving signal (ν_FAS_) measured by bi-directional axial transmission (BDAT) bone ultrasound in the radius and tibia is significantly lower in XLH patients compared to healthy controls. This finding is in agreement with the hypothesis that compromised cortical bone tissue properties in XLH patients (i.e., lower tissue mineralization, hypo-mineralized holes, increased pore size, higher porosity) result in a reduction of the compressional wave velocities compared to age-matched controls. The range of values and age dependency of the ν_FAS_ is in agreement with reference values obtained by a comparable axial transmission device (34).

Musculoskeletal symptoms associated with bone deformities and skeletal muscle fatigue significantly contribute to the burden of disease in children and adults with XLH: More than 80% of children and adults report abnormal gait and skeletal pain underlining the disease impact on quality of life (5). On radiographs, typical imaging signs of rickets and osteomalacia including poor fracture healing can be observed (3, 35). Previously, DXA studies have shown increased aBMD in adult XLH patients, especially in the axial skeleton (6, 7, 14–16). Bearing in mind that DXA is a projectional technique of low image resolution, which is further affected by anthropometric indices, DXA is clearly unable to provide a full insight into bone quality in XLH. Specifically, bone microarchitecture including Ct.Po cannot be measured by DXA. Histological studies have shown mineralization defects (14, 26), but necessitate an invasive procedure. Bone quality can be assessed non-invasively by HR-pQCT. Microarchitectural changes in both the cortical and trabecular bone have been reported in HR-pQCT studies (16, 19). QUS represents another option for non-invasive assessment of bone quality not involving ionizing radiation, which is an advantage especially in children. To our best knowledge, no studies have investigated QUS in XLH so far.

Previous studies showed impaired ultrasound properties (e.g., sound velocity and attenuation) in children with growth disturbances and bone disorders (22). The mineralization defects of the tissue matrix in XLH patients are believed to create a “vicious cycle”, i.e., softer tissue leads to more strain, overstimulation of osteocytes, and increased formation of deformed bone (26). This explains why XLH patients have equal or even increased levels of BMD measured by DXA or HR-pQCT compared to healthy subjects, despite softened cortical bone, as measured by BDAT. All of our XLH study patients underwent treatment at the time of the study, but the compromised mechanical competence of the cortical bone tissue in XLH patients was clearly visible both, in the clinical phenotype and by means of reduced ν_FAS_ values in the tibia and radius bones. ν_FAS_ is a surrogate measure of three cortical bone properties contributing to bone stiffness and strength, i.e., Ct.Th, tissue matrix stiffness, and Ct.Po (23). For a thickness-wavelength ratio smaller than 0.5, ν_FAS_ represents a guided wave propagation mode, which is slower than the compressional wave mode measured for larger thickness-wavelength ratios. For our measurements, ν_FAS_ values can be considered to represent the velocity of compressional waves. The absolute deviation of individual values from age and gender-matched controls may be a suitable indicator for the severity of the mineralization deficit. However, the individual parameters contributing to the reduction of ν_FAS_ in XLH patients remain subject to further studies, which should also integrate more recent techniques that are able to measure Ct.Th, Ct.Po, and speed of sound independently (24, 36, 37) as well as second-generation HR-pQCT, which can provide site-matched measurements at central shaft regions. As BDAT and other ultrasound-based quantitative bone imaging modalities use no ionizing radiation, they would be ideal for the treatment monitoring of children and young adults with bone diseases.

As the number of HR-pQCT sub-study participants was very small, results have to be interpreted with caution. Also, the scanning position is harder to be set in XLH patients due to the different morphology of the physis compared to healthy individuals. In concordance with Colares Neto et al. and Shanbhogue et al. the children and adolescents with XLH in our study exhibited lower trabecular number, higher trabecular spacing and higher trabecular network inhomogeneity, though not statistically significant. In the ultra-distal tibia, trabecular thickness (Tb.Th) was significantly higher in XLH patients compared to healthy controls (*p* = 0.021). Colares Neto et al. have reported greater Tb.Th in the radius of XLH patients than in controls and after stratification by age higher Tb.Th in children than in adults. In contrast, they did not find any significant differences in Tb.Th in the tibia (16). Shanbhogue et al. did not find any significant differences in Tb.Th between adult XLH patients and healthy controls in neither the radius nor the tibia (19). Our predominantly young study population may explain this finding.

Quantitative bone ultrasound may be a complementary tool to monitor treatment response and optimize drug dosing. Although axial transmission is not an imaging modality and can therefore not provide information about the degree of bone deformity or visualize the growth plate, it measures cortical bone properties in long bones, which is not accessible with conventional imaging modalities.

Treatment options for XLH are currently expanding beyond phosphate and vitamin D. A novel treatment with burosumab, a FGF-23 antibody, has shown superiority to conventional treatments regarding improvement of rickets and linear growth (38). Future studies should look further into the pathomechanism of ν_FAS_ reduction in XLH patients and how to use this information for treatment monitoring—be it the traditional phosphate and vitamin D approach or burosumab. The ultimate goal would be to prevent skeletal deformities in XLH patients. Quantitative bone ultrasound, in particular BDAT may also represent a promising tool for radiation free monitoring of other pediatric bone disorders with cortical phenotypes, including primary, or secondary osteoporosis.

The main limitation of our study is a low sample size as a result of the low prevalence of XLH. We lacked power to evaluate associations between ν_FAS_ and structural parameter as well as sex differences, because of the small number of subjects in this study. On the other hand, strengths of our study are that it is the first study to investigate QUS in XLH patients and healthy controls. Another strength is the age range of our study participants, which were predominantly pediatric, an age group that is most often referred to clinicians in specialized treatment centers.

In conclusion, we could prove that ν_FAS_ measured using BDAT ultrasound can be used as surrogate parameter for decreased matrix stiffness and/or increased Ct.Po in XLH. Pediatric and young adult patients with XLH exhibited a significant reduction in ν_FAS_ as compared to healthy controls. BDAT or other novel quantitative bone ultrasound modalities could potentially be used for radiation-free treatment monitoring in XLH to improve treatment guidance and to evaluate novel therapeutic options.

## Data Availability Statement

The raw data supporting the conclusions of this article will be made available by the authors, without undue reservation.

## Ethics Statement

The studies involving human participants were reviewed and approved by Ethics Committee of the Medical University of Vienna. Written informed consent to participate in this study was provided by the participants' legal guardian/next of kin.

## Author Contributions

AR, GH, PP, JP, and KR contributed to the conception and design of the study. AR, AB-M, RK, MK, and PF organized the patient recruitment and site management in Vienna. KR and JS organized volunteer recruitment, performed BDAT measurements and data evaluation. MW, SM, and AR organized the database. MW, KR, and JS performed the statistical analysis. SM, AR, and JP wrote the first draft of the manuscript. KR, AB-M, MK, and JS wrote sections of the manuscript. All authors contributed to manuscript revision, proof-reading, and approved the submitted version.

## Conflict of Interest

The authors declare that the research was conducted in the absence of any commercial or financial relationships that could be construed as a potential conflict of interest.

## Supplementary Material

Figure S1 Comparative illustration of paired BDAT/HR-pQCT data.
