# Supplementary figures and images for: Decreased Compressional Sound Velocity Is an Indicator for Compromised Bone Stiffness in X-Linked Hypophosphatemic Rickets (XLH)
Source: Front Endocrinol (Lausanne). 2020 Jun 9;11:355. doi: 10.3389/fendo.2020.00355 (PMC7296046; doi:10.3389/fendo.2020.00355)

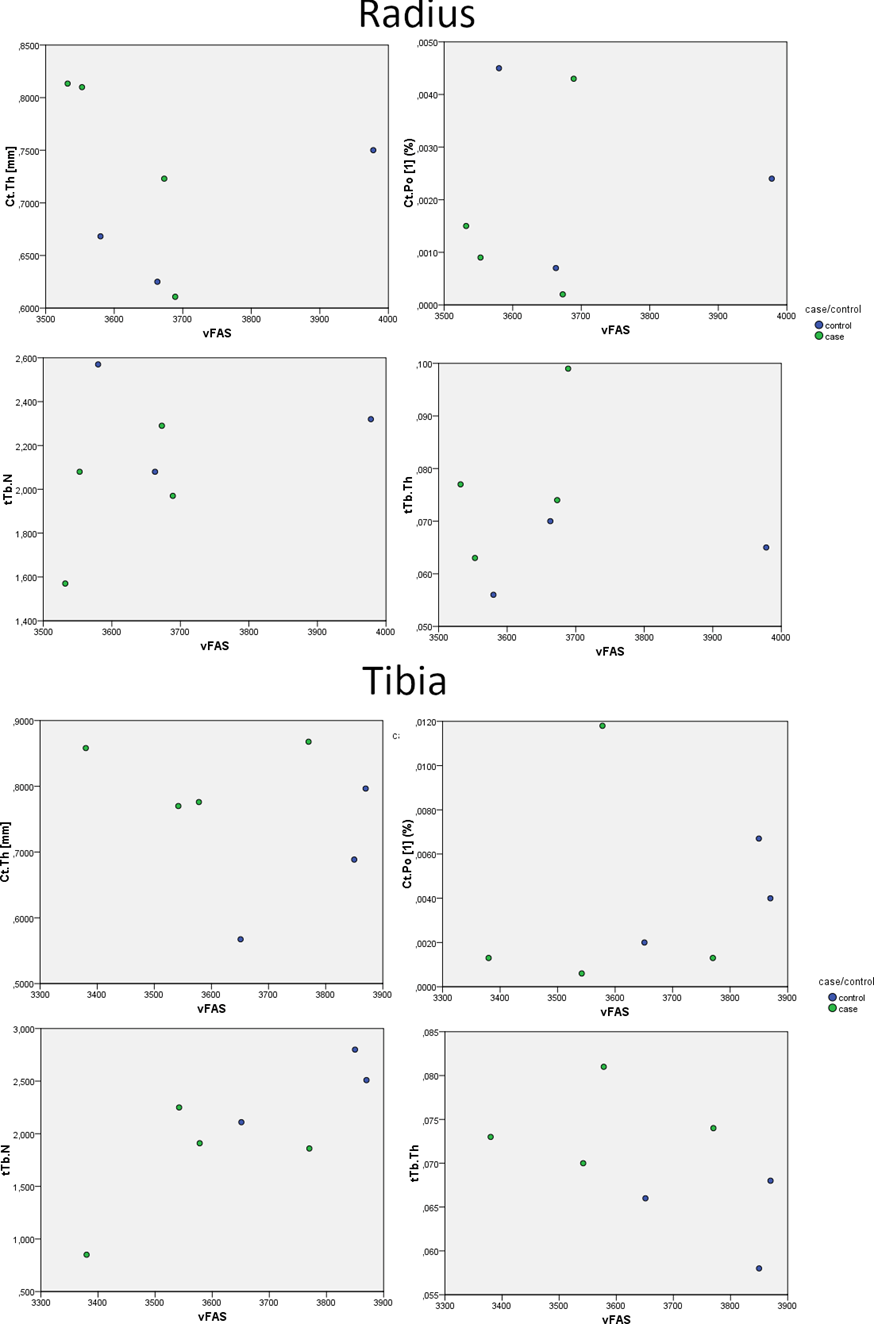

Supplement: Figure S1 — Comparative illustration of paired BDAT/HR-pQCT data. [file Image_1.TIFF]
